# Differential actions of orexin receptors in brainstem cholinergic and monoaminergic neurons revealed by receptor knockouts: implications for orexinergic signaling in arousal and narcolepsy

**DOI:** 10.3389/fnins.2013.00246

**Published:** 2013-12-20

**Authors:** Kristi A. Kohlmeier, Christopher J. Tyler, Mike Kalogiannis, Masaru Ishibashi, Morten P. Kristensen, Iryna Gumenchuk, Richard M. Chemelli, Yaz Y. Kisanuki, Masashi Yanagisawa, Christopher S. Leonard

**Affiliations:** ^1^Department of Drug Design and Pharmacology, Faculty of Health and Medical Sciences, University of CopenhagenCopenhagen, Denmark; ^2^Department of Physiology, New York Medical CollegeValhalla, NY, USA; ^3^Howard Hughes Medical Institute, University of Texas Southwestern Medical CenterDallas, TX, USA

**Keywords:** laterodorsal tegmental nucleus, dorsal raphe nucleus, locus coeruleus, whole-cell patch-clamp recording, Ca^2+^ signaling

## Abstract

Orexin neuropeptides influence multiple homeostatic functions and play an essential role in the expression of normal sleep-wake behavior. While their two known receptors (OX_1_ and OX_2_) are targets for novel pharmacotherapeutics, the actions mediated by each receptor remain largely unexplored. Using brain slices from mice constitutively lacking either receptor, we used whole-cell and Ca^2+^ imaging methods to delineate the cellular actions of each receptor within cholinergic [laterodorsal tegmental nucleus (LDT)] and monoaminergic [dorsal raphe (DR) and locus coeruleus (LC)] brainstem nuclei—where orexins promote arousal and suppress REM sleep. In slices from OX^−/−^_2_ mice, orexin-A (300 nM) elicited wild-type responses in LDT, DR, and LC neurons consisting of a depolarizing current and augmented voltage-dependent Ca^2+^ transients. In slices from OX^−/−^_1_ mice, the depolarizing current was absent in LDT and LC neurons and was attenuated in DR neurons, although Ca^2+^-transients were still augmented. Since orexin-A produced neither of these actions in slices lacking both receptors, our findings suggest that orexin-mediated depolarization is mediated by both receptors in DR, but is exclusively mediated by OX_1_ in LDT and LC neurons, even though OX_2_ is present and OX_2_ mRNA appears elevated in brainstems from OX^−/−^_1_ mice. Considering published behavioral data, these findings support a model in which orexin-mediated excitation of mesopontine cholinergic and monoaminergic neurons contributes little to stabilizing spontaneous waking and sleep bouts, but functions in context-dependent arousal and helps restrict muscle atonia to REM sleep. The augmented Ca^2+^ transients produced by both receptors appeared mediated by influx via L-type Ca^2+^ channels, which is often linked to transcriptional signaling. This could provide an adaptive signal to compensate for receptor loss or prolonged antagonism and may contribute to the reduced severity of narcolepsy in single receptor knockout mice.

## Introduction

Orexin-A and Orexin-B, also called hypocretin-1 and -2, are two hypothalamic neuropeptides (De Lecea et al., 1998; Sakurai et al., 1998) that influence feeding, metabolism, arousal, reward, and stress substrates in the CNS (for review, see Tsujino and Sakurai, 2009). The orexin system plays a particularly important role in the normal expression of waking and sleep since its disruption underlies the sleep disorder narcolepsy with cataplexy in humans (Peyron et al., 2000; Thannickal et al., 2000) and produces a narcolepsy phenotype with unstable behavioral states, sleep attacks and cataplexy-like motor arrests in animals (Chemelli et al., 1999; Lin et al., 1999; Hara et al., 2001; Willie et al., 2003; Beuckmann et al., 2004; Mochizuki et al., 2004; Kalogiannis et al., 2011).

Orexin actions are mediated by two G-protein coupled receptors (Sakurai et al., 1998), termed orexin-1 (OX_1_) and orexin-2 (OX_2_) receptors, which have partly overlapping and widespread expression patterns in the CNS (Trivedi et al., 1998; Hervieu et al., 2001; Marcus et al., 2001). These receptors are attractive targets for the development of a range of novel therapeutic agents with potential for treating sleep disorders, obesity, stress-related disorders, and addiction. The first orexin-related drugs to appear will be the dual orexin receptor antagonists (DORAs) for the treatment of insomnia (Uslaner et al., 2013; Winrow and Renger, 2013), but there is significant interest in developing single orexin receptor-specific antagonists (SORAs). However, much remains unknown about the cellular actions of each receptor and the behavioral consequences of activation or inhibition of each receptor at their many targets.

In this study, we focus on mesopontine cholinergic [laterodorsal tegmental nucleus (LDT)] and monoaminergic [dorsal raphe (DR) and locus coeruleus (LC)] neurons, which participate in a spectrum of functions that include the control of arousal and sleep (Jones, 2005; Brown et al., 2012), the maintenance of motor activity and muscle tone during arousal (Jacobs and Fornal, 1993; Michelsen et al., 2007), the mediation of stress related actions and adaptation (Lowry et al., 2005; Valentino and Van Bockstaele, 2008) and the stimulation of motivated behavior via projections to midbrain dopamine systems (Maskos, 2008; Mena-Segovia et al., 2008). These structures, and especially the LC, receive substantial orexinergic innervation (Peyron et al., 1998; Chemelli et al., 1999; Nambu et al., 1999), and orexin terminals have been shown to synapse upon tyrosine hydroxylase immunoreactive neurons in LC (Horvath et al., 1999), cholinergic neurons in the LDT (Cid-Pellitero and Garzón, 2011) and DR neurons (Del Cid-Pellitero and Garzón, 2011). Evidence from *in-situ* hybridization studies in rat (Marcus et al., 2001) indicate that moderate levels of OX_1_ mRNA are expressed in LDT and DR while especially high levels of OX_1_ mRNA are expressed in the LC. These studies also found moderate levels of OX_2_ mRNA levels in the DR with lower levels in the LDT and LC. Consistent with this innervation pattern and receptor distribution, exogenously applied orexins directly depolarize LDT (Burlet et al., 2002; Kohlmeier et al., 2008) and related PPT (Kim et al., 2009) neurons, along with DR (Brown et al., 2002; Liu et al., 2002; Kohlmeier et al., 2008) and LC neurons (Horvath et al., 1999; Ivanov and Aston-Jones, 2000; Li et al., 2002; Hoang et al., 2003; Murai and Akaike, 2005) partly, by activating a cation current. Orexin-A has also been shown previously to have a distinct modulatory role in the LDT and DR by augmenting the Ca^2+^ influx mediated by L-type Ca^2+^ channels (Kohlmeier et al., 2008). However, the roles played by each receptor in these actions are unknown.

Early studies of orexin receptors using heterologous expression found that both receptors stimulate Ca^2+^-release from intracellular stores (Sakurai et al., 1998; Smart et al., 1999) and activate phospholipase C (PLC) (Lund et al., 2000; Holmqvist et al., 2002), suggesting they are *G*_q_ coupled receptors. More recent studies indicate these receptors can couple to multiple G-proteins and therefore may utilize more diverse signaling cascades, however, much less is known about the signaling targets of native orexin receptors (for review see Kukkonen and Leonard, 2013). Evidence from studies of brain slices or freshly dissociated neurons indicate that native orexin receptors mediate neuronal depolarization from resting membrane potential by activating three classes of effectors (for review see Leonard and Kukkonen, 2013): closure of K^+^ channels (Ivanov and Aston-Jones, 2000; Hwang et al., 2001; Bayer et al., 2002; Grabauskas and Moises, 2003; Hoang et al., 2003, 2004; Ishibashi et al., 2005; Bisetti et al., 2006), forward activation of the electrogenic Na^+^/Ca^2+^ exchanger (Eriksson et al., 2001; Wu et al., 2002, 2004; Burdakov et al., 2003), activation of a cation current (Brown et al., 2002; Yang and Ferguson, 2002, 2003; Murai and Akaike, 2005; Kohlmeier et al., 2008) and can elevate intracellular [Ca^2+^] (Van Den Pol et al., 1998; Van Den Pol, 1999; Uramura et al., 2001; Xu et al., 2002; Kohlmeier et al., 2004, 2008; Ishibashi et al., 2005). Only limited information is available about the particular receptors mediating these actions, especially since receptor overlap is common and subtype-specific antagonists are lacking. Conclusions about native receptor function has often relied upon the relative potencies of orexin-A and -B, based on the high potency of orexin-A for both receptors and the ~10-fold lower potency of orexin-B for OX_1_ expressed in CHO cells (Sakurai et al., 1998). However, agonist-based receptor determinations have numerous potential confounds (see Leonard and Kukkonen, 2013) including differing stability of agonists, differing receptor levels and the possibility of biased agonism, as has been proposed for expressed orexin receptors (Putula et al., 2011).

To avoid these potential limitations, we sought to determine the actions of each receptor in LDT, DR, and LC neurons using a genetic dissection approach: We examined the actions by each receptor using whole-cell recording and Ca^2+^ imaging methods in brain slices from knockout mice constitutively lacking either receptor thereby allowing us to determine the functional capacity of each receptor in isolation. This revealed that OX_1_ and OX_2_ have both convergent and unique functions in LDT, DR, and LC neurons. These findings have implications for understanding the cellular functions of orexin receptors, the behavioral consequences of orexin signaling at these loci and some consequences for using receptor specific antagonists as therapeutics.

## Materials and methods

All procedures complied with National Institutes of Health (US) and institutional guidelines (New York Medical College) for ethical use of animals and were approved by the New York Medical College Institutional Animal Care and Use Committee (IACUC).

### Animals and genotyping

Brain slices for whole cell recordings were prepared from 14 to 32 day old C57BL6, background control (orexin receptor wild-type; OxrWT), OX^−/−^_2_, OX^−/−^_1_, and OX^−/−^_1_/OX^−/−^_2_ double knockout (DKO) mice. In those cases where calcium imaging was being performed with the cell permeant fura-2AM, mice aged 9–15 days old were utilized. Both male and female mice were used in this study and receptor knockout mice were the offspring of breeders that were both homozygous for the null alleles on mixed C57BL6 and 129SvEv genetic backgrounds. The OxrWT mice were bred from the wild-type progeny of the OX^+/−^_2_ parents used to make the OX^−/−^_2_ mice. The OxrWT, OX^−/−^_2_, OX^−/−^_1_, and DKO mice have been described previously (Willie et al., 2003; Kalogiannis et al., 2011; Mieda et al., 2011).

To confirm genotypes, tail biopsies were obtained during slice preparation and subsequently analyzed by PCR. One set of primers were used to determine if the mouse was either a wild-type or knockout for each orexin receptor. The three primers for OX_1_ consisted of a common primer (5′-CTCTTTCTCCACAGAGCCCAGGACTC-3′), a knockout primer (5′-TGAGCGAGTAACAACCCGTCGGATTC-3′) and a wild-type primer (5′gCAAGAATGGGTATGAAGGGAAGGGC-3′). The expected product sizes were ~320 base pairs for the wild-type allele and ~500 base pairs for the knockout allele. The three primers for OX_2_ consisted of a common primer (5′-CTGGTGCAAATCCCCTGCAAA-3′), a knockout primer (5′-GGTTTTCCCAGTCACGACGTTGTA-3′) and a wild-type primer (5′-AATCCTTCTAGAGATCCCTCCTAG-3′). The expected product sizes were ~620 base pairs for wild-type allele and ~300 base pairs for the knockout allele. These two sets of primers for different orexin receptors were processed separately. PCR amplification consisted of denaturation at 95°C for 8 min followed by 35 cycles of 94°C (30 s), 62°C (30 s) and 72°C (1 min), followed by one cycle at 72°C for 10 min. The result of each PCR reaction was then separated using a 2% agarose gel, and the PCR product was visualized with ethidium bromide.

### Brain slices and electrophysiology

Brain slices (250 μm) were prepared using a Leica vibratome (VT1000S) in ice-cold artificial cerebrospinal fluid (ACSF) which contained (in mM): 121 NaCl, 5 KCl, 1.2 NaH_2_PO_4_, 2.7 CaCl_2_, 1.2 MgSO_4_, 26 NaHCO_3_, 20 dextrose, 4.2 lactic acid and was oxygenated by bubbling with carbogen (95% O_2_ and 5% CO_2_). Slices containing the LDT, DR, or LC were incubated at 35°C for 15 min in oxygenated ACSF, and were then stored at room temperature in continuously oxygenated ACSF, until they were utilized for recordings.

For recording, slices were placed in a submersion chamber on a fixed-stage microscope (Olympus BX50WI) and superfused (1–2 ml/min) with ACSF (23 ± 2°C). Regions for recording were chosen from within the boundaries of the LDT, DR, or LC nuclei determined using a 4X objective and brightfield illumination. Neurons were then visualized with a video camera and DIC optics using a 40X water immersion objective (Olympus; NA 0.8). Large to medium sized multipolar neurons were selected for gigaseal recording in voltage clamp or current clamp mode using an Axoclamp 2A or Axopatch 2A or 2B amplifier (Axon Instruments). Borosilicate patch pipettes (2–5 MOhms; cat number 8050, AM systems) containing a solution of (in mM) 144 K-Gluconate, 0.2 EGTA, 3 MgCl_2_, 10 HEPES, 0.3 NaGTP, 4 Na_2_ATP. Biocytin (0.1%) with the Na-GTP added to the pipette solution just before use.

### Drugs and experimental solutions

Normal ACSF contained (in mM) 124 NaCl, 5 KCl, 1.2 NaH_2_PO_4_, 2.7 CaCl_2_, 1.2 MgSO_4_, 26 NaHCO_3_ and 10 dextrose (295–305 mOsm). To block voltage-gated sodium channels and fast synaptic potentials, the ACSF (DABST-containing) contained the ionotropic receptor antagonists DNQX (15 μ M, Sigma), APV (50 μM, Sigma), bicuculline (10 μM, Sigma), and strychnine (2.5 μ M, Sigma) with TTX (500 nM, Alomone). To measure the average orexin-A mediated post-synaptic current and its I-V relation in LDT and DR neurons, CsCl (2 or 3 mM) was added to the DABST to block H-current and a low Ca^2+^ ACSF was used to inhibit voltage-gated Ca^2+^ currents. [Ca^2+^] was buffered to <20 μ M by the addition of 2.7 mM EGTA (calculated with Patcher's Power Tools XOP for Igor Pro). Orexin-A (Sigma, USA; Phoenix Pharmaceuticals, USA; American Peptides, USA; Peptide international, USA) was dissolved in deionized water or physiological saline in 1 mM aliquots and frozen (−80°C). Aliquots were dissolved in ACSF to a final concentration of 300 nM immediately before use. The L-type Ca^2+^ channel antagonist nifedipine and agonist, Bay-K-8644 (Bay-K; Sigma, USA) were dissolved in DMSO to a stock concentration of 10 mM and delivered at the final concentration of 10 μ M in ACSF. Bisindolylmaleimide I, HCl (Calbiochem, EMD Biosciences) was dissolved in DMSO to a stock concentration of 5 mM. On the day of experiments it was diluted in TTX-ACSF to a final concentration of 1 μ M and applied for 5 min prior to application of orexin. Final dilutions of these drugs were made immediately before application and light exposure was minimized throughout preparation and application.

### Ca^2+^ imaging

Fluorescence related to intracellular calcium concentration was measured from neurons in slices that had been either individually filled with bis-fura-2 from patch pipettes or bulk-loaded with fura-2AM. The patch solution for Ca^2+^ imaging contained the potassium salt of bis-fura-2 (50 μ M, Molecular Probes) dissolved in a solution containing (in mM) 144 K-gluconate, 3 MgCl_2_, 10 HEPES, 0.3 NaGTP, and 4 Na_2_ATP. Biocytin (0.1%) or biotinylated Alexa-594 (25 μ M; Invitrogen) was included in all experiments for cell identification following slice fixation.

For bulk loading neurons with fura-2, slices from young mice (P6-P17) were incubated in ACSF containing 15 μ M fura-2AM (Molecular Probes) prepared from a 3.3 mM stock of fura-2AM in DMSO. Slices were incubated for 30 min at 36°C in a small volume equilibrated with Carbogen (5% CO_2_/95% O_2_). Slices were then transferred to the recording chamber and rinsed for at least 30 min to ensure de-esterification and temperature equilibration. After locating a recording region in the LDT, DR, or LC, individual cells were imaged with the 40× water immersion lens.

Ca^2+^ transients were monitored by measuring the emission at 515 nm resulting from excitation of fura-2 with 380 nm (F_380_; 71000 Chroma fura-2 filter set) from a shuttered 75W Xenon light source. Optical recordings were made using a back illuminated, frame-transfer, cooled CCD camera system (EEV 57 chip, Micromax System, Roper Scientific) that was controlled with custom software (TI Workbench) running on a Mac OS computer. Images were either acquired discontinuously (every 1–4 s) with the shutter closed between images (600 ms exposure) or continuously (~50 ms/frame), with the shutter open for the entire epoch. Changes in intracellular calcium concentration were inferred from changes in delta F/F (dF/F) where F is the fluorescence at rest within a ROI following subtraction of background fluorescence. dF is the change in fluorescence following subtraction of the average F prior to stimulation. dF/F was usually corrected for photobleaching. Since rises in [Ca^2+^] produce a decrease in F_380_ with fura-2, all dF/F measures are inverted so positive-going traces indicate elevation of [Ca^2+^]_*i*_.

### Immunocytochemistry

Recorded neurons were identified as cholinergic, serotonergic or noradrenergic by using conventional immunocytochemistry following slice fixation in 4% paraformaldehyde, cryoprotection and re-sectioning at 40 μm on a freezing microtome. Filled neurons were visualized with avidin-Texas Red or Alexa-594 biocytin. Cholinergic neurons in the LDT selectively co-localize the enzyme neuronal nitric oxide synthase (nNOS; Vincent et al., 1983) and were identified by immunolabeling for nNOS (1:400 rabbit polyclonal, Sigma, Cat N7280). Serotonergic neurons in the DR were identified by immunolabeling for tryptophan hydroxylase (TpH; 1:400 sheep polyclonal, Abcam, 3907 and Covance, PSH-327P). Catecholamine neurons in the LC were identified by immunolabeling for tyrosine hydroxylase (TH; 1:1000, rabbit polyclonal, Abcam, Cat 112). For immunofluorescence, primary antibodies were visualized with FITC or Alexa 488-labeled secondary antibodies. Following washing and mounting, cleared sections were imaged using appropriate filter sets with a CCD camera (Coolsnap, Roper Scientific or QICam, QImaging) mounted on an epi-fluorescence microscope (BX60; Olympus). For immunohistochemistry primary antibodies were visualized with biotinylated secondary antibodies and the tissue was processed with a Vector ABC kit as per the manufacturer's instructions and reacted with Sigma's FastDAB kit for 5 min (Sigma-Aldrich, St. Louis, MO). The precipitation reaction was stopped with three rises of PBS and the sections were mounted on slides and coverslipped.

### RNA isolation from whole brainstem

To isolate RNA from whole brainstems, C57BL6, OX^−/−^_1_, OX^−/−^_2_, DKO, and 129SvEv mice were anesthetized and decapitated as for brain slices. The brainstems were then rapidly dissected from the whole brain starting ~5 mm anterior to the superior colliculus to the medulla, and the cerebellum was removed. The brainstems were then flash-frozen in liquid nitrogen. Total RNA was isolated from the frozen tissue using the RNeasy Lipid Tissue Mini kit (Qiagen). RNA quality and quantity was determined by the A260/A280 ratio from the spectrophotometer.

Standards were generated from cDNA isolated from the C57BL6 brainstem. The cDNA was loaded with primers of the gene of interest and amplified using a conventional PCR for 35 cycles. The product was then run on a gel to determine the specificity of the amplification. If there was a single band of the predicted size, the concentration of the PCR product was determined through spectrophotometry and serially diluted. This technique was utilized with all the primers to create scales unique for each gene of interest. These scales were used to correlate target mRNA fluorescence to sample starting concentration. Scales were created for OX_1_ and for each of the two splice variants of OX_2_ identified in mouse: OX_2_α and OX_2_β (Chen and Randeva, 2004; Chen et al., 2006). OX_1_ mRNA scales ranged from 1.8 × 10^−2^ to 1.8 × 10^−5^μ g/ml. OX_2_α mRNA scales ranged from 7.7 × 10^−2^ to 7.7 × 10^−5^ μ g/ml. OX_2_β mRNA scale ranged from 9.0 × 10^−3^ to 9.0 × 10^−6^ μg/ml.

### Light cycler real time PCR

A 20 μ l volume of the RNA solution, with ~1 μg of total RNA from each sample was reverse transcribed using random primers and the Improm II Reverse Transcription (RT) kit (Promega). The samples were incubated with random primers for 5 min at 70°C, 5 min at 4°C and 1 h at 37°C followed by 70°C for 15 min. The RT product was then aliquoted and stored at −20°C. Two microliters of the RT product were loaded with SYBR green I reagent, 25 mM MgCl_2_ and primers to a final volume of 20 μ l. Primers for OX_1_, OX_2_α, and OX_2_β were loaded at a constant concentration (10 μ M) for their respective runs (Invitrogen). The following sequences were used to amplify the genes of interest: OX_1_ forward: 5′-TGCCGCCAACCCTATCATCT-3′ reverse: 5′-GTGACGGTGGTCAGCACGAC-3′ (which corresponds to pubmed genbank NM_198959, 1364-1547); OX_2_α forward: 5′-GAGACAAGCTTGCAGCACTGAG-3′ reverse: 5′-TGAGTCGGGTATCCTCATCATAG-3′; OX_2_β forward: 5′-GAGACAAGCTTGCAGCACTGAG-3′ reverse: 5′-GGTCGGTCAATGTCCAATGTTC-3′ (Chen and Randeva, 2004; Chen et al., 2006). Each sample was loaded in duplicate (Roche, Lightcycler RT-PCR). Light cycler protocols for mRNA quantification consisted of denaturation at 95°C (485 s), cycling 40 times at 94°C (5 s), 64°C (10 s), and 76°C (14 s). The same amount of total RNA was loaded into each capillary tube for the lightcycler. The measured starting amount of target mRNA was then normalized by the amount of total RNA loaded. The mRNA/total RNA ratios were subsequently compared between samples obtained from C57BL6, OX^−/−^_1_, OX^−/−^_2_, DKO, and 129SvEv mice. Lightcycler products were then visualized with ethidium bromide in 2% agarose gels to confirm the presence of the RT-PCR target.

### Data analysis

Data analysis and figure preparation was done using Igor Pro (Wavemetrics) software. Differences between means were determined by paired or unpaired Student's *t*-test or a One Way ANOVA using MS excel or DataDesk 6 software (Data Description, Inc). Non-parametric comparisons were conducted utilizing Chi-Square analysis using excel or DataDesk 6 or the Kolmogorov-Smirnov test using Igor Pro. Numerical results are reported as mean ± SEM.

## Results

### Principal neurons in the LDT, DR, and LC appear normal in orexin receptor null mice

**Figure 1:**
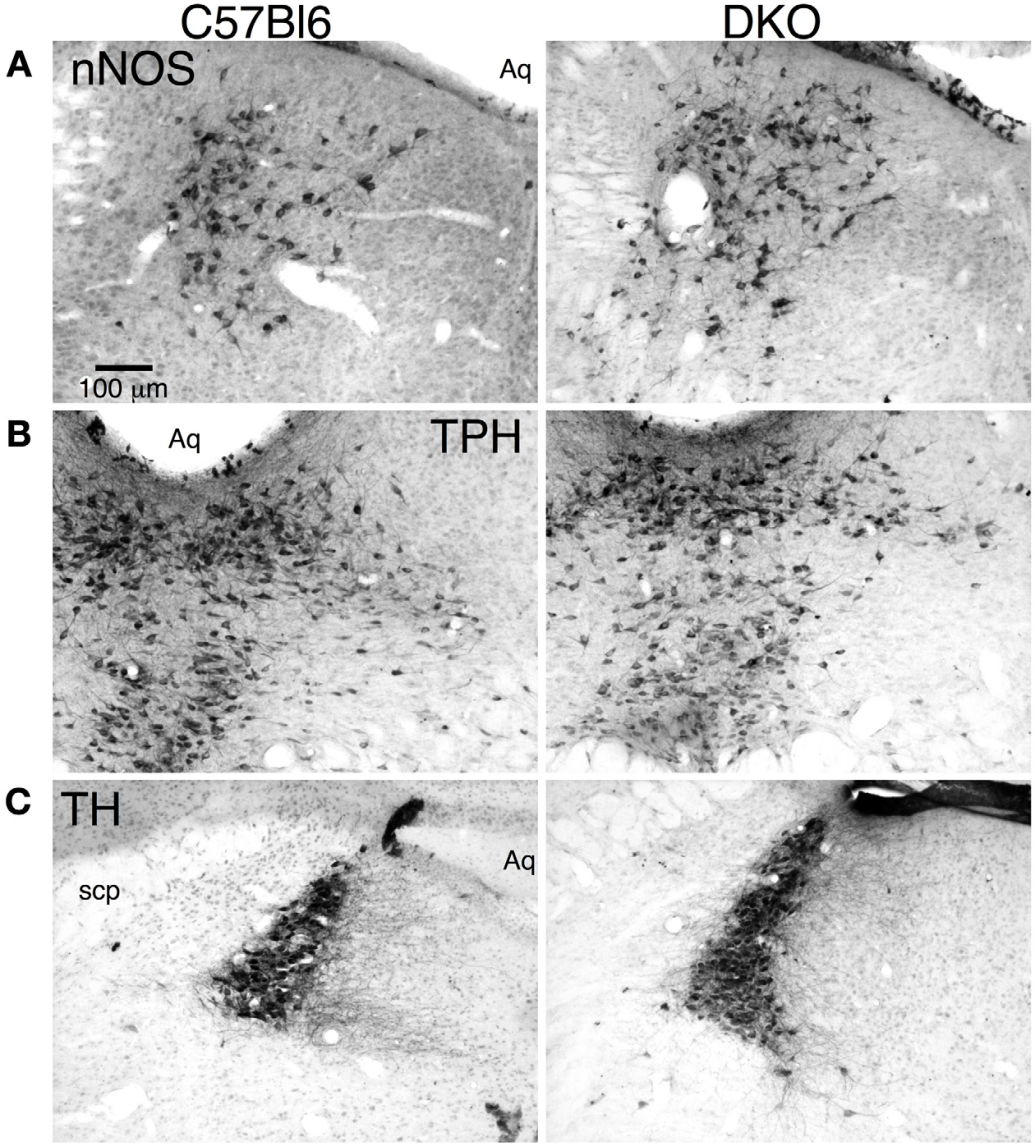
**Double orexin receptor knockout (DKO) mice showed grossly normal distributions of brainstem cholinergic and monoaminergic neurons. (A)** nNOS immunolabeled sections through the caudal LDT stained with DAB from a C57BL6 mouse (left) and a DKO mouse (right). **(B)** TpH immunolabeled sections through the caudal DR stained with DAB from a C57BL6 mouse (left) and a DKO mouse (right). **(C)** TH immunolabeled sections through the LC stained with DAB from a C57BL6 mouse (left) and a DKO mouse (right). Abbreviations: Aq, Aqueduct; scp, superior cerebellar peduncle.

To determine if the absence of orexin receptors resulted in gross anomalies in the development of brainstem cholinergic and monoaminergic nuclei, we inspected immunohistochemically stained tissue sections through the LDT, DR, and LC. We examined nNOS, TpH, and TH staining in sections from C57BL6 mice (*n* = 2) and from mice lacking one or both receptors (*n* = 2 for each genotype). Figure 1 shows comparable tissue sections through the LDT, DR, and LC from a C57BL6 mouse (left column) and a DKO mouse (right column) stained for nNOS (top row), TpH (middle row), and TH (bottom row). Immunoreactivity appeared highly specific and the expected pools of labeled neurons were observed in sections from each genotype. No gross differences were observed in distribution of stained neurons or in the range of cell shapes in any of the receptor knockouts. This suggests that mesopontine cholinergic and monoaminergic neurons will be found in their expected locations and will be encountered at about the same rate in brain slice experiments from normal and receptor knockout mice. Of course, this qualitative observation does not rule out quantitative differences that might be present between knockout and wild type mice (see Kalogiannis et al., 2010; Valko et al., 2013).

### Orexin-a produces comparable noisy inward current in slices from C57BL6 and hybrid background control mice

In our previous studies of orexin actions on LDT and DR neurons, we conducted whole-cell recordings and Ca^2+^ imaging using slices made from C57BL6 mice (Burlet et al., 2002; Kohlmeier et al., 2004, 2008). Since receptor null mice were on C57BL6/129SvEv hybrid background, we initially examined the ability of a bath application of orexin-A (300 nM) to evoke an inward current from a holding potential of −60 mV in LDT and DR neurons in slices from C57BL6 and background control mice (OxrWT). As expected, orexin-A activated a noisy inward current in both nuclei and this current appeared identical in both strains. In the LDT, we recorded 16 neurons from 13 C57BL6 mice and 7 neurons from 6 OxrWT mice. There was no difference in the mean current (C57BL6: −19.1 ± 2.4 pA; OxrWT: −15.2 ± 3.1 pA; *P* = 0.38) and there was no difference in the distribution of current amplitudes, including the three C57BL6 and two OxrWT neurons that didn't respond (Kolmogorov-Smirnov test, *P* = 0.24). In the DR, we recorded 31 neurons from 22 C57BL6 mice and 16 neurons from 8 OxrWT mice. There was no difference in the mean current from cells that responded to orexin (C57BL6: −36.3 ± 2.9 pA; OxrWT: −36.9 ± 3.2 pA; *P* = 0.89) and there was no difference in the distributions, including the one C57BL6 and two OxrWT neurons that didn't respond (Kolmogorov-Smirnov test, *P* = 1). This suggests that the differences in genetic background of C57BL6 and hybrid mice has little influence on the expression of orexin currents.

### Activation of OX_1_ produces inward current and enhanced Ca^2+^ transients in LDT, DR, and LC neurons

**Figure 2:**
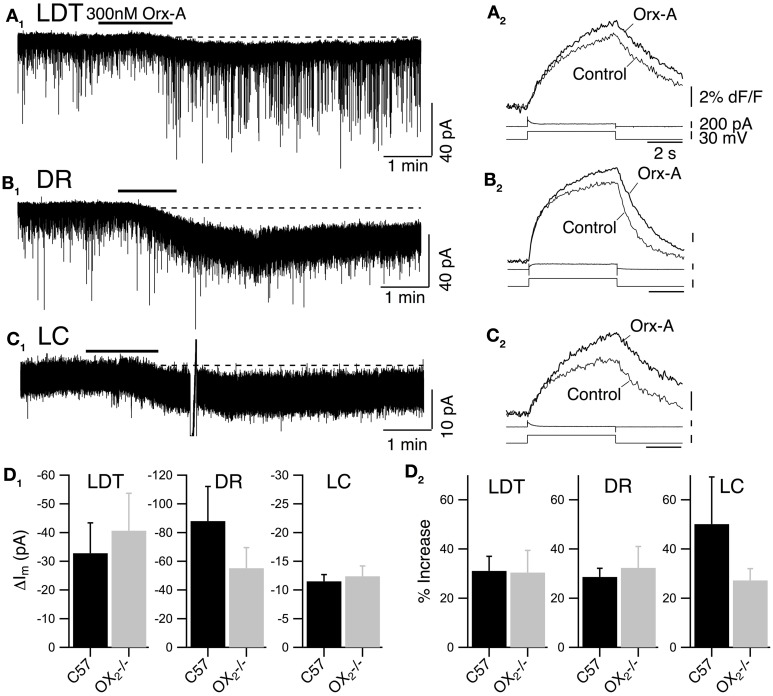
**Recordings from OX^−/−^_2_ slices indicate that OX_1_ is sufficient for orexin-mediated excitation and enhancement of voltage-dependent Ca^2+^ transients in LDT, DR, and LC neurons. (A)** Whole-cell voltage clamp recording from LDT neurons in OX^−/−^_2_ slices showed that 300 nM orexin-A (Orx-A) still evoked an inward current with increased sEPSCs [holding potential: −60 mV; **(A_**1**_)**; normal ACSF] and also augmented the Ca^2+^ transients evoked by 5 s voltage-steps from −60 to −30 mV [**(A_**2**_)**; DABST-containing ACSF]. **(B)** Voltage clamp recordings from DR neurons in OX^−/−^_2_ slices showed that orexin-A evoked an inward [holding potential: −60 mV; **(B_**1**_)**; normal ACSF] and augmented Ca^2+^ transients [**(B_**2**_)**; DABST-containing ACSF]. **(C)** Voltage clamp recordings from LC neurons in OX^−/−^_2_ slices showed that orexin-A evoked an inward current [holding potential: −60 mV; **(C_**1**_)**; normal ACSF] and augmented Ca^2+^ transients [**(C_**2**_)**; DABST-containing ACSF]. In (**A_1_,B_1_,C_1_**), the horizontal bar indicates time of 300 nM orexin-A superfusion. In (**A_2_,B_2_,C_2_**), top traces show somatic Ca^2+^-dependent fluorescence (dF/F); Middle traces show whole-cell current; Bottom traces show membrane voltage. Calibration bars indicate 2% dF/F, 200 pA, 30 mV, and 2 s. **(D)** Neither the orexin-evoked inward current nor the orexin-enhanced voltage-dependent Ca^2+^ transients was different in neurons from OX^−/−^_2_ slices compared to wild-type slices. Comparison by nuclei of the mean ± SEM of the post-synaptic inward current [**(D_**1**_)**; LDT and DR recorded in low-Ca^2+^ DABST-containing ACSF with Cs; LC recorded in DABST-containing ACSF] and mean ± SEM of the Ca^2+^ transient enhancement [**(D_**2**_)**; DABST-containing ACSF] produced by orexin-A in neurons recorded from OX^−/−^_2_ slices and C57BL6 slices under the same conditions.

To test the ability of OX_1_ to sustain orexin signaling in the LDT, DR, and LC, we recorded from slices made from OX^−/−^_2_ mice and compared the orexin responses obtained in these knockouts with those in slices obtained from C57BL6 mice (Figure 2). In each nucleus, we examined the ability of a bath application of orexin-A (300 nM) to evoke an inward current from a holding potential of −60 mV and to enhance the Ca^2+^ influx produced by a voltage-step from −60 to −30 mV.

In LDT neurons recorded from OX^−/−^_2_ mice, orexin-A evoked a slowly developing inward current, often with an increase in frequency and amplitude of sEPSPs (Figure 2A_1_; normal ACSF) that appeared quite similar to the responses previously described in nNOS+ LDT neurons (Burlet et al., 2002). We did not analyze the increase in sEPSC frequency in detail, but we compared the average magnitude of the slowly developing inward orexin current recorded from a holding potential of −60 mV (low-Ca^2+^ DABST-containing ACSF with Cs). This post-synaptic depolarizing current was −40.7 ± 13.0 pA (*n* = 7) which was not statistically different from the inward current measured from LDT neurons in slices from C57BL6 mice recorded under identical conditions (32.8 ± 10.6 pA; *n* = 8; *P* > 0.05; Figure 2D_1_). Orexin-A (300 nM) also enhanced the Ca^2+^-transient evoked by a 5 s voltage-jump from −60 to −30 mV by 30.4 ± 6.4% (*n* = 9/12) in neurons from OX^−/−^_2_ mice (Figure 2A_2_; DABST-containing ACSF). The magnitude of this Ca^2+^ transient enhancement was also not different from that measured in LDT neurons from C57BL6 mice (31.1 ± 7.7%; *n* = 9/13; *P* > 0.05; Figure 2D_2_).

In DR neurons recorded in slices from OX^−/−^_2_ mice, orexin A (300 nM) also evoked a slow inward current (Figure 2B_1_; normal ACSF) as observed previously (Brown et al., 2002; Liu et al., 2002; Kohlmeier et al., 2008). These inward currents were not accompanied by increases in sEPSCs, as expected from a previous study (Haj-Dahmane and Shen, 2005). The average amplitude of the orexin-evoked slow inward current was −55.2 ± 14.4 pA (*n* = 8; low-Ca^2+^ DABST-containing ACSF with Cs). This was not statistically different from the orexin-evoked current measured from DR neurons in slices from C57BL6 mice under the same conditions (88.0 ± 24.1 pA; *n* = 7; *P* > 0.05; Figure 2D_1_). In the voltage-step paradigm, orexin-A (300 nM) also enhanced the Ca^2+^-transient evoked by steps from −60 to −30 mV by 32.3 ± 8.7% (*n* = 13/18) in neurons from OX^−/−^_2_ mice (Figure 2A_2_; DABST-containing ACSF) as reported in wild-type DR neurons (Kohlmeier et al., 2008). This enhancement in neurons from OX^−/−^_2_ mice was also not different from that measured in DR neurons from C57BL6 mice (28.6 ± 3.5, *n* = 9/14; *P* > 0.05; Figure 2D_2_) recorded under the same conditions.

In LC neurons from OX^−/−^_2_ mice, orexin-A (300 nM) produced a small, slow inward current (Figure 2C_1_; normal ACSF). The average current evoked by orexin-A was 11.4 ± 1.2 pA (*n* = 28; DABST-containing ACSF) and was not different from the average current measured from LC neurons in slices from C57BL6 mice under the same conditions (12.4 ± 1.8 pA; *n* = 12; *P* > 0.05; Figure 2D_1_). Orexin-A (300 nM) also produced a strong enhancement of the Ca^2+^ transient evoked by a voltage step from −60 to −30 mV (Figure 2C_2_; DABST-containing ACSF) in LC neurons. This enhancement was on average 27.2 ± 4.8% (*n* = 11/17) and was not different from that obtained from LC neurons from C57BL6 mice (50.19 ± 19.1%; *n* = 6/8; *P* > 0.05; Figure 2D_2_) recorded under the same conditions. Collectively, these data indicate that OX_1_ is sufficient to mediate two normal actions of orexin on LDT, DR, and LC neurons: (1) post-synaptic activation of a slowly developing inward current and (2) the enhancement of voltage-dependent Ca^2+^ influx.

**Figure 3:**
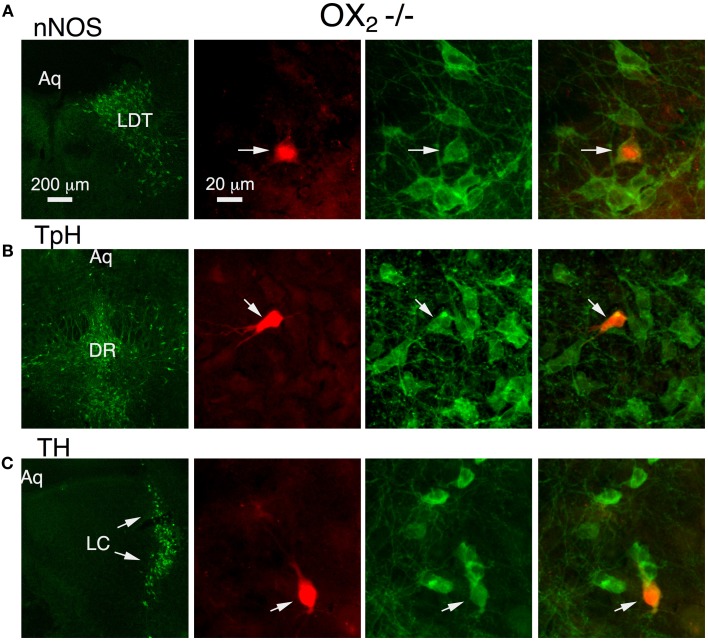
**Stimulation of OX_1_ alone is sufficient to produce inward currents and augmented voltage-dependent Ca^2+^ transients in nNOS+ LDT neurons (A), TpH+ DR neurons (B), and TH+ LC neurons (C)**. Left column illustrates low-power fluorescent micrographs of the recorded slices immunostained and visualized with an Alexa-488-label (green) for nNOS in LDT **(A)**, TpH in DR **(B)**, and TH in the LC **(C)**. The second column illustrates a higher-power image of the recorded and red fluorescently labeled neuron (arrow; Alexa 594) in each nucleus. The third column shows the same field with Alexa 488 visualized. The right column merges the Alexa 488 and 594 images and indicates that each recorded neuron was immunopositive for nNOS, TpH, and TH, respectively.

To determine if OX_1_ mediated these actions in principal neurons from each nucleus, we processed brain slices from OX^−/−^_2_ mice for immunocytochemistry to identify the transmitter phenotype of recorded neurons. In the LDT, we confirmed that OX_1_ activation produced an inward current in six nNOS+ neurons and that OX_1_ activation enhanced the voltage-step evoked Ca^2+^ transient in four nNOS+ neurons. In the DR, we confirmed that OX_1_ activation produced an inward current in nine TpH+ neurons and enhanced the voltage-step evoked Ca^2+^ transient in four TpH+ neurons. Similarly, in the LC, we confirmed that OX_1_ activation produced an inward current in 24 TH+ neurons and enhanced the voltage-step evoked Ca^2+^ transient in 2/4 TH+ neurons. Figure 3 illustrates examples of an nNOS+ neuron recorded in the LDT (Figure 3A), a TPH+ neuron recorded in the DR (Figure 3B) and a TH+ neuron recorded in the LC. Each of these neurons showed an OX_1_-mediated inward current and an enhancement of the Ca^2+^ transient evoked by voltage steps from −60 to −30 mV. From these data we conclude that OX_1_ is sufficient to mediate major direct orexin actions on principal cells of these nuclei.

### OX_1_ activates a noisy cation current in LDT and DR neurons and enhances Ca^2+^ transients mediated by L-type Ca^2+^ channels in LDT, DR, and LC neurons

**Figure 4:**
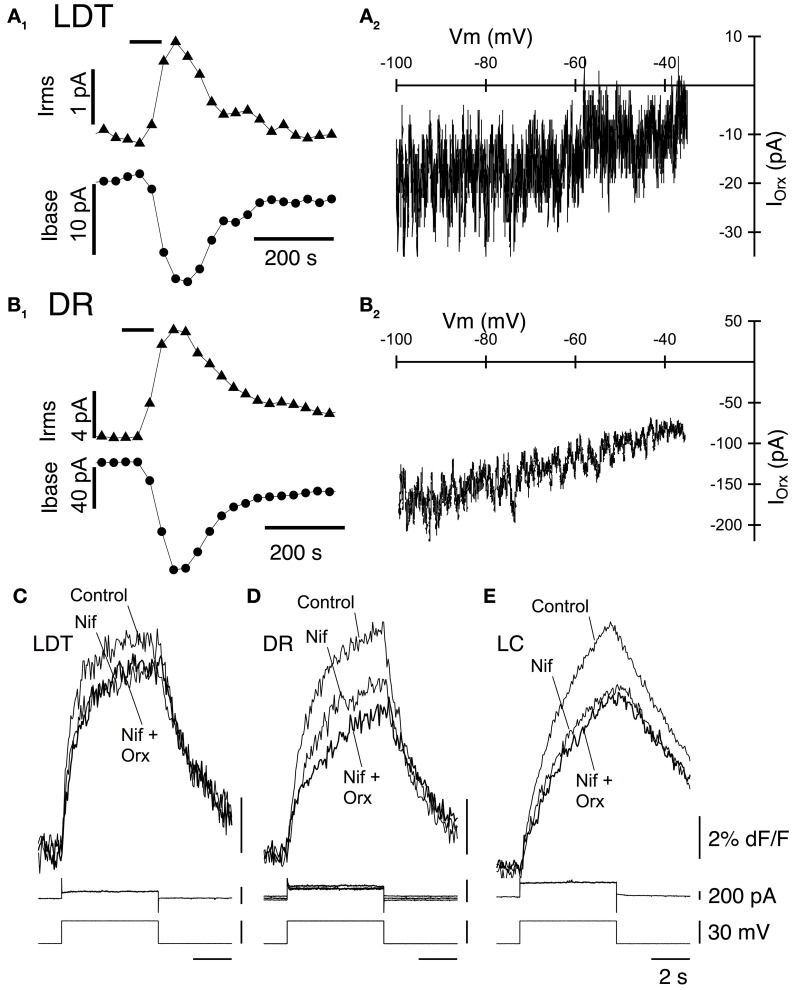
**Stimulation of OX_1_ alone activates a noisy cation current in LDT and DR neurons and enhance voltage-dependent Ca^2+^ transients mediated by L-type Ca^2+^ channels in LDT, DR, and LC neurons. (A_1_)** Holding current (at −60 mV; Ibase; bottom trace) and membrane current noise (Irms; top trace) were measured every 30 s starting before bath application of 300 nM orexin-A (horizontal bar) from LDT neurons in OX^−/−^_2_ slices. Orexin produced an inward shift in holding current that was accompanied by an increase in current noise. **(A_2_)**. The I-V curve of the orexin-evoked inward current (I_Orx_) was obtained by subtracting the membrane current produced by a voltage ramp between −100 and −35 mV prior to orexin-A application from that obtained during the peak of the orexin-A-evoked inward current. These currents were similar to those obtained from LDT neurons in C57BL6 slices. **(B_1_)** Orexin-A (300 nM) has a similar, but larger effect on the holding current (Ibase, bottom) and membrane current noise (Irms, top) in DR neurons recorded in OX^−/−^_2_ slices. **(B_2_)** The I-V relation for the orexin-evoked inward current (I_Orx_) in a DR neuron from an OX^−/−^_2_ slice was similar that that observed in DR neurons from C57BL6 slices. **(C,D,E)**. The L-channel antagonist, nifedipine (10 μM) attenuated the Ca^2+^-transients evoked by voltage-steps from −60 to −30 mV in LDT **(C)**, DR **(D)**, and LC **(E)** neurons from OX^−/−^_2_ slices and completely blocked the enhancement of these transients by orexin-A (300 nM). Top traces show somatic Ca^2+^-dependent fluorescence (dF/F); Middle traces show whole-cell current; Bottom traces show membrane voltage. Calibration bar labels in **(E)**, also apply to **(C)** and **(D)**.

As noted above, previous studies of LDT and DR neurons indicate that a noisy non-selective cation current is an important effector mediating the slow membrane depolarization produced by orexin-A. We therefore examined the change in membrane noise and the current-voltage relation of the current evoked in LDT and DR neurons from OX^−/−^_2_ mice to determine if OX_1_ is competent to activate a similar current. Since current evoked by orexin-A in LC neurons was quite small in our recordings, and since they did not show a similar increase in noise, we did not further characterize their orexin currents. In both LDT (Figure 4A_1_) and DR (Figure 4B_1_) neurons from OX^−/−^_2_ mice, orexin-A (300 nM) produced an inward current that was accompanied by a large increase in membrane current noise, similar to that reported in wild-type mice (Kohlmeier et al., 2008). The membrane current noise increased by 118.4 ± 59.3% in LDT neurons (*n* = 8) and by 307 ± 87.0% in DR neurons (*n* = 7) which was not different from values in LDT (86.8 ± 31.3%, *n* = 8; *P* > 0.05) and DR (380.5 ± 85.2%; *n* = 7; *P* > 0.05) from C57BL6 mice. Similarly, the I-V relation for the orexin mediated current in LDT (Figure 4A_2_) and DR neurons (Figure 4B_2_) from OX^−/−^_2_ mice was computed from voltage ramps between −100 and −35 mV. It appeared roughly linear over this range as previously described for C57BL6 neurons in these nuclei (Kohlmeier et al., 2008) suggesting that OX_1_ activates the same channel or channels that are activated in C57BL6 mice.

In LDT and DR neurons from C57BL6 mice, the orexin mediated enhancement of voltage-dependent Ca^2+^ transients produced by voltage-steps from −60 to −30 mV is produced almost entirely by the enhancement of Ca^2+^ transients generated by L-type Ca^2+^ channels (Kohlmeier et al., 2008). We therefore tested whether the L-channel antagonist nifedipine (10 μM), also occludes this enhancement in LDT, DR, and LC neurons in slices from OX_2_ mice. Nifedipine alone, attenuated the Ca^2+^ transient produced by a voltage-step from −30 to −60 mV in LDT (Figure 4C), DR (Figure 4D), and LC (Figure 4E) neurons indicating that activation of L-type channels contribute to these transients. In the presence of nifedipine, orexin failed to significantly enhance the calcium transient in all cells examined within the LDT, DR, or LC (LDT: 9% reduction in the transient, *P* > 0.05. *n* = 4; DR: 5.2% reduction in the transient, *P* > 0.05, *n* = 4; LC: 1.8% increase in the transient, *P* > 0.05, *n* = 4). These data suggest that the Ca^2+^ transients augmented by OX_1_ signaling are mediated by enhanced influx via L-type Ca^2+^ channels. Thus, activation of OX_1_ alone is sufficient to activate a noisy cation current in LDT and DR neurons and to enhance Ca^2+^ transients which appear generated by L-type Ca^2+^ channels in the LDT, DR, and LC.

### Activation of OX_2_ excites DR neurons and enhances Ca^2+^ transients in LDT, DR, and LC neurons

**Figure 5:**
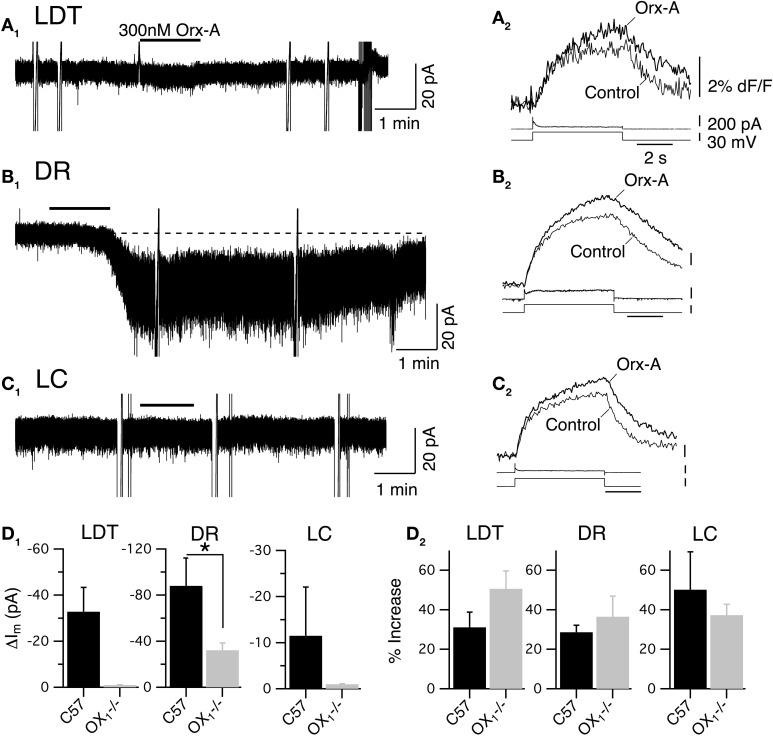
**Stimulation of OX_2_ alone is sufficient to produce an inward current in DR neurons and to enhance voltage-dependent Ca^2+^ transients in LDT, DR, and LC neurons. (A)** Whole-cell voltage clamp recordings obtained from LDT neurons in OX^−/−^_1_ slices showed that 300 nM orexin-A (Orx-A) failed to evoke a current at −60 mV **(A_**1**_)** but augmented voltage-dependent Ca^2+^ transients evoked by 5 s voltage-steps from −60 to −30 mV **(A_**2**_). (B)** Voltage clamp recordings obtained from DR neurons in OX^−/−^_1_ slices showed that orexin-A evoked both a noisy inward current (holding potential: −60 mV; **B_**1**_)** and augmented voltage-dependent Ca^2+^ transients **(B_**2**_). (C)** Voltage clamp recordings obtained from LC neurons in OX^−/−^_1_ slices showed that orexin-A failed to evoke an inward current (holding potential: −60 mV; **C_**1**_**) but augmented voltage-dependent Ca^2+^ transients **(C_**2**_)**. In **(A_**1**_,B_**1**_,C_**1**_)**, the horizontal bar indicates time of 300 nM orexin-A superfusion. In **(A_**2**_,B_**2**_,C_**2**_)**, Top traces show somatic Ca^2+^-dependent fluorescence (dF/F); Middle traces show whole-cell current; Bottom traces show membrane voltage. Calibration bars indicate 2% dF/F, 200 pA, 30 mV and 2 s. **(D)** The orexin-A evoked inward current was absent in LDT and LC neurons and was significantly smaller in DR neurons from OX^−/−^_1_ slices than in DR neurons from C57BL6 slices (D_**1**_; ^*^P < 0.05). In contrast, the magnitude of the Ca^2+^ transient enhancement produced by orexin-A was the same in LDT, DR, and LC neurons from OX^−/−^_1_ slices compared to those recorded in C57BL6 slices.

To determine whether activation of OX_2_ alone can influence membrane currents and Ca^2+^ transients in LDT, DR, and LC neurons, we examined the action of orexin-A in slices prepared from mice lacking OX_1_. Whole-cell recordings from LDT (*n* = 14) and LC (*n* = 45) neurons revealed that in that absence of OX_1_, 300 nM orexin-A failed to induce a detectable inward current at a holding potential of −60 mV (Figures 5A_1_,C_1_). In contrast, orexin-A application to DR neurons resulted in significant inward current with a large increase in membrane noise (Figure 5B_1_). Nevertheless, the average peak inward current elicited by orexin-A in DR neurons (32.2 ± 6.3 pA; *n* = 8) was significantly lower than observed in control recordings from DR neurons in slices from C57BL6 mice (88.0 ± 24.1 pA; *n* = 7; *P* < 0.05; Figure 5D_1_).

**Figure 6:**
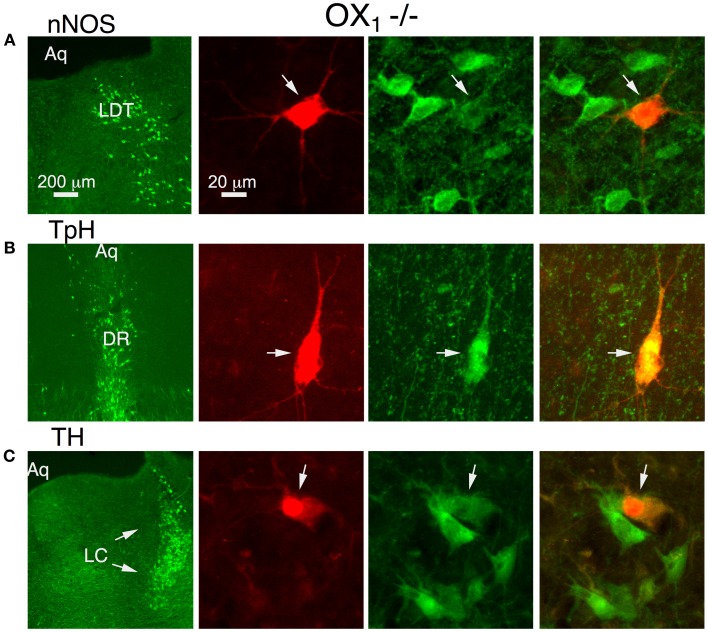
**Orexin augments voltage-dependent Ca^2+^ transients in nNOS+ LDT neurons, TpH+ DR neurons, and TH+ LC neurons in slices from OX^−/−^_1_ mice**. Orexin-A augmented the voltage-dependent Ca^2+^ transient in identified neurons. Left column illustrates low-power fluorescent micrographs of the recorded slices immunostained with Alexa-488 (green) for nNOS in LDT **(A)**, TpH in DR **(B)**, and TH in the LC (**C** arrows). The second column illustrates a higher-power image of the recorded and fluorescently labeled neurons (Alexa-594) from each nucleus. The third column shows the same field with Alexa-488 visualized. The right column shows the images merged, revealing that each recorded neuron was immunopositive for nNOS, TpH, and TH, respectively.

Despite being unable to stimulate inward currents in LDT and LC neurons from OX^−/−^_1_ mice, orexin-A was effective at enhancing Ca^2+^ transients evoked by voltage-jumps from −60 to −30 mV in LDT, DR and LC neurons (Figures 5A_2_,B_2_,C_2_). In fact, on average the magnitude of the enhancement in the absence of OX_1_ (LDT: 50.6 ± 9.0%, *n* = 7/17; DR: 36.5 ± 10.4, *n* = 11/18; LC: 37.3 ± 5.5, *n* = 8/13) was as large as observed in control neurons from C57BL6 mice (Figure 5D_2_; *P* > 0.05 for each cell type). In a some recordings, we were able to verify that the recorded neurons were nNOS+ in the LDT (*n* = 6), TpH+ in the DR (*n* = 9), and TH+ in the LC (*n* = 13; Figure 6). This confirmed that OX_2_ alone is insufficient to excite neurons in the LDT and LC at subthreshold membrane potentials but is sufficient to do so in TpH+ DR neurons. In spite of this, OX_2_ activation is able to augment the step-activated Ca^2+^ transients in principal neurons of each of these nuclei. This implies that released orexin would enhance Ca^2+^ influx during persistent activity in each nucleus, even in OX_1_ null mice.

**Figure 7:**
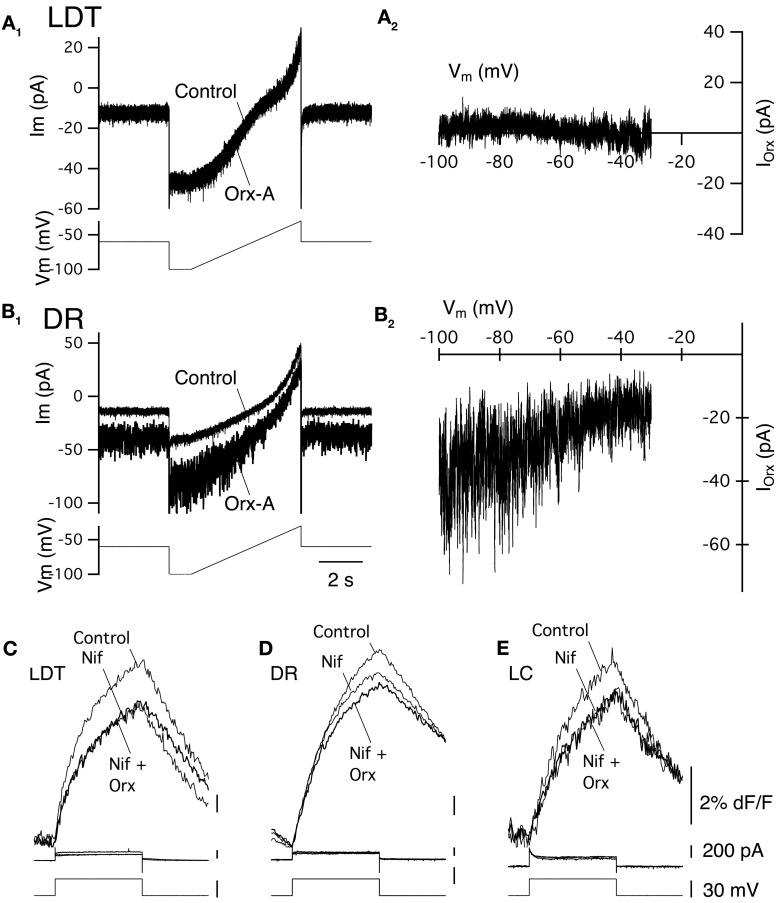
**Stimulation of OX_2_ alone activate a noisy cation current in DR neurons and enhance Ca^2+^ transients mediated by L-type Ca^2+^ channels in LDT, DR, and LC neurons. (A_1_)** Whole-cell voltage clamp currents (upper traces) from an LDT neuron in an OX^−/−^_1_ slice recorded before and after bath superfusion with orexin-A (Orx-A, 300 nM). These currents were produced by the voltage-ramp in the bottom trace (−100 to −35mV). Orexin failed to produce an inward shift in holding current in LDT neurons from OX^−/−^_1_ mice. **(A_2_)** The difference between the currents in **(A_1_)** plotted as a function of membrane voltage (Vm) shows the I-V relation. No orexin current was detectable throughout the voltage range studied. **(B_1_)** Orexin-A (300 nM) produced an inward shift in holding current and a large increase in membrane current noise in DR neurons recorded in OX^−/−^_1_ slices. **(B_2_)** The I-V relation for the difference current from **(B_1_)** appeared nearly linear and was characteristic of the cation current observed in DR neurons from C57BL6 slices. **(C,D,E)** The L-channel antagonist, nifedipine (Nif, 10 µM) attenuated the Ca^2+^-transients evoked by voltage-steps from −60 to −30 mV in LDT **(C)**, DR **(D)**, and LC **(E)** neurons from OX^−/−^_1_ slices and prevented the enhancement of these transients by orexin-A (300 nM). Top traces show somatic Ca^2+^-dependent fluorescence (dF/F); Middle traces show whole-cell current; Bottom traces show membrane voltage. Calibration bar labels in **(E)**, also apply to **(C,D)**.

We also examined the I–V relation between −100 and −30 mV in LDT and DR neurons. This confirmed that no observable sub-threshold currents were activated by orexin-A in LDT neurons (Figures 7A_1_,A_2_) and that the noisy inward current activated in DR neurons was approximately linear over this voltage range, having similar characteristics to the non-selective cation current observed in C57BL6 DR neurons (Figures 7B_1_,B_2_). Similarly, we determined whether L-type Ca^2+^ channels are a target of OX_2_ by testing whether nifedipine occluded the orexin-A enhancement of Ca^2+^ transients (Figures 7C,D,E). In LDT, DR, and LC neurons obtained from mice lacking OX_1_, nifedipine blocked the enhancement produced by orexin-A (300 nM) application. In the presence of nifedipine, orexin-A application produced a 0.6 ± 0.4% increase in LDT (*n* = 4), a 4.0 ± 2.1% increase in DR (*n* = 5) and a 3.6 ± 3.3% increase in LC neurons (*n* = 6). These changes were not significantly different from zero (*P* > 0.05) and all of these differences were significantly less than changes produced in neurons from C57BL6 mice (*P* < 0.05). Thus, activation of OX_2_ alone is sufficient to activate a noisy cation current in DR neurons but not in LDT or LC neurons. Despite this, OX_2_ activation is sufficient to enhance Ca^2+^ transients in the LDT, DR, and LC. Moreover, the magnitude of this enhancement was comparable to the enhancement measured in LDT, DR, and LC neurons from C57BL6 mice and also appear mediated by L-type Ca^2+^ channels.

### OX_1_ signaling is dominant in the LDT and LC but is more evenly shared by both receptors in the DR

**Figure 8:**
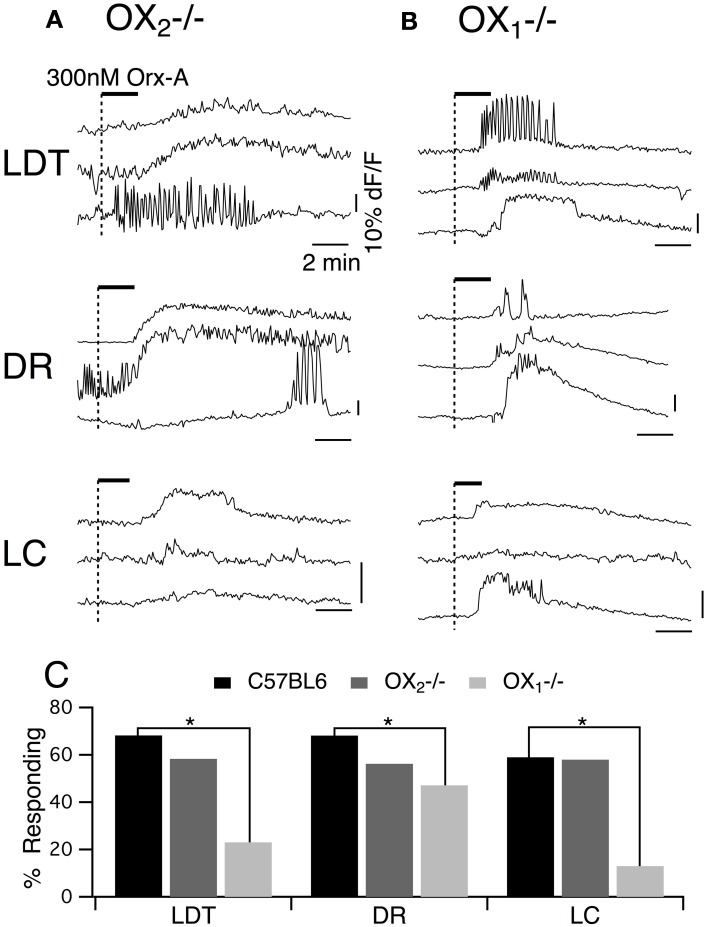
**Orexin-A stimulates a larger fraction of LDT, DR, and LC cells via OX_1_ than via OX_2_. (A)** Orexin-A (Orx-A, 300 nM) evoked Ca^2+^ transients in LDT, DR, and LC neurons in slices from OX^−/−^_2_ mice that were bulk-loaded with fura-2AM. **(B)** Orexin-A (300 nM) also evoked Ca^2+^ transients in LDT, DR, and LC neurons in slices from OX^−/−^_1_ mice that were bulk-loaded with fura-2AM. The magnitude of the transients evoked by either receptor was not different from that evoked in slices from C57BL6 mice (see text). **(C)** In slices from C57BL6 mice, 60–70% of fura-2AM labeled cells in LDT (*n* = 173), DR (*n* = 113), and LC (*n* = 98) were activated by orexin. The percentage of cells imaged that responded to orexin-A in slices from OX^−/−^_2_ mice (LDT: *n* = 60; DR: *n* = 96; LC: *n* = 43) was not statistically different from that in C57BL6 slices. A significantly smaller percentage of cells imaged in each nucleus was activated by orexin in slices from OX^−/−^_1_ mice (LDT: *n* = 113; DR:*n* = 87; LC: *n* = 82). Horizontal bars above the traces indicate application of orexin-A (300 nM). Calibration bars indicate 10% dF/F and 2 min in each panel. ^*^P < 0.05.

To obtain a better estimate of the fraction of neurons activated by each orexin receptor, we utilized fura-2AM loading of slices obtained from mice lacking each receptor (Figure 8). Under these conditions, orexin-A (300 nM) evokes Ca^2+^ transients by both depolarization and subsequent activation of a voltage-dependent Ca^2+^ influx and by specific enhancement of the Ca^2+^ transient evoked by L-type Ca^2+^ channel activation (Kohlmeier et al., 2004, 2008). As expected from whole-cell Ca^2+^ transient measurements, 300 nM orexin-A evoked Ca^2+^ transients in slices obtained from mice lacking OX_2_ (Figure 8A) and mice lacking OX_1_ (Figure 8B). We found that in each genotype and nucleus, these transients recapitulated the range of temporal profiles we observed in DR and LDT slices from C57BL6 mice (Kohlmeier et al., 2004). Moreover, when the magnitude of the “plateau” type Ca^2+^ responses, which we could confidently measure, were compared, the transients from knockout slices were not smaller than the transients from C57BL6 slices. In OX_2_ null tissue, the average plateau responses were 11.9 ± 1.8% in LDT (*n* = 13); 9.9 ± 1.9% in DR (*n* = 18); and 12.1 ± 2.4% in LC (*n* = 12), which were not different (*P* > 0.05) from responses measured in slices from C57BL6 mice (11.0 ± 2.2% in LDT, *n* = 29; 13.8 ± 2.6% in DR, *n* = 49; and 12.7 ± 2.3% in LC, *n* = 24). Similarly, in OX_1_ null slices, the average plateau response were not different (*P* > 0.05) from those in C57BL6 slices (OX_1_ null responses: 12.0 ± 4.3% in LDT, *n* = 12; 16.1 ± 2.5% in DR, *n* = 15; and 15.1 ± 5.1% in LC, *n* = 5). However, the likelihood of encountering orexin-A responsive cells (Figure 8C) was much lower in slices from OX_1_ null mice in both the LDT and LC. The proportion of fura-2AM labeled cells responding to orexin was only 20% in LDT (*n* = 113) and 10% in LC (*n* = 82) compared to estimates of 60–70% responding in slices from C57BL6 mice. Interestingly, in the DR from OX_1_ null mice, the fraction of responders was only reduced to 47.1% from about 70% in the C57BL6 mice. Presumably this high percent responding in the DR reflects the ability of OX_2_ to drive depolarizations in DR neurons unlike in LDT and LC neurons.

### OX_1_ and OX_2_ signaling is attenuated by PKC inhibition

**Figure 9:**
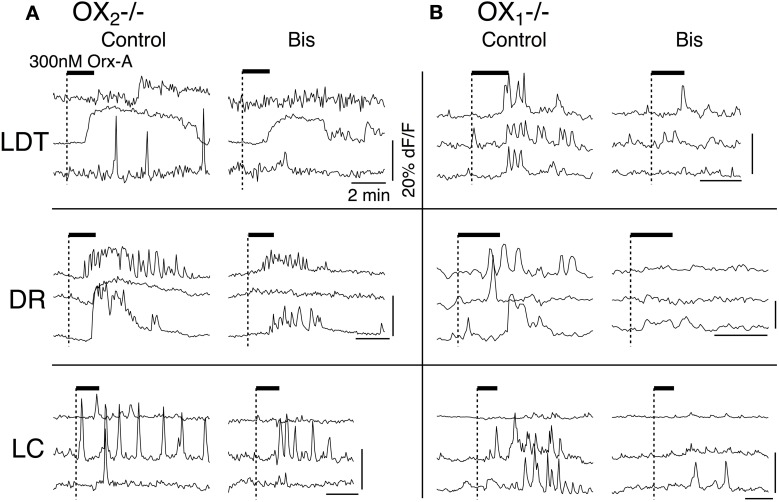
**Inhibition of PKC by Bis I attenuated Ca^2+^-transients evoked by activation OX_1_ or OX_2_. (A)** Ca^2+^ transients in LDT, DR, and LC neurons in slices from OX^−/−^_2_ mice were attenuated by prior application of Bis I. **(B)** Similarly, Ca^2+^ transients in LDT, DR, and LC neurons in slices from OX^−/−^_1_ mice were attenuated by prior application of Bis I. Horizontal bars above the traces indicate application of orexin-A (300 nM). Calibration bars indicate 20% dF/F and 2 min in each panel.

In the next series of experiments we examined whether the PKC inhibitor Bis I attenuates the Ca^2+^ transients evoked by activity of one or both receptors. In these experiments, we first obtained control responses with bath application of orexin-A (300 nM) from OX^−/−^_2_ or OX^−/−^_1_ slices containing LDT, DR, or LC (Figures 9, left column in **A,B**). The slices were then superfused with ACSF containing bis I (1 μM; Bis) for 20 min, to inhibit PKC. This completely attenuated the orexin-mediated enhancement of L-type Ca^2+^ transients but did not block the orexin mediated depolarization in all LDT and DR neurons (Kohlmeier et al., 2008). Here, we found that the Ca^2+^ transients evoked in each nucleus were strongly attenuated by Bis in slices from both OX_2_ and OX_1_ null mice (Figures 9, right column in **A,B**). The average amplitude of plateau responses was reduced by 62.0 ± 7.7% in slices from OX_2_ null mice (*n* = 34) and by 59.3 ± 8.3 in slices from OX^−/−^_1_ mice (*n* = 25). Thus, Ca^2+^ transients evoked by activation of either orexin receptor involves PKC signaling.

### Orexin actions in the LDT, DR, and LC require the two known orexin receptors

**Figure 10:**
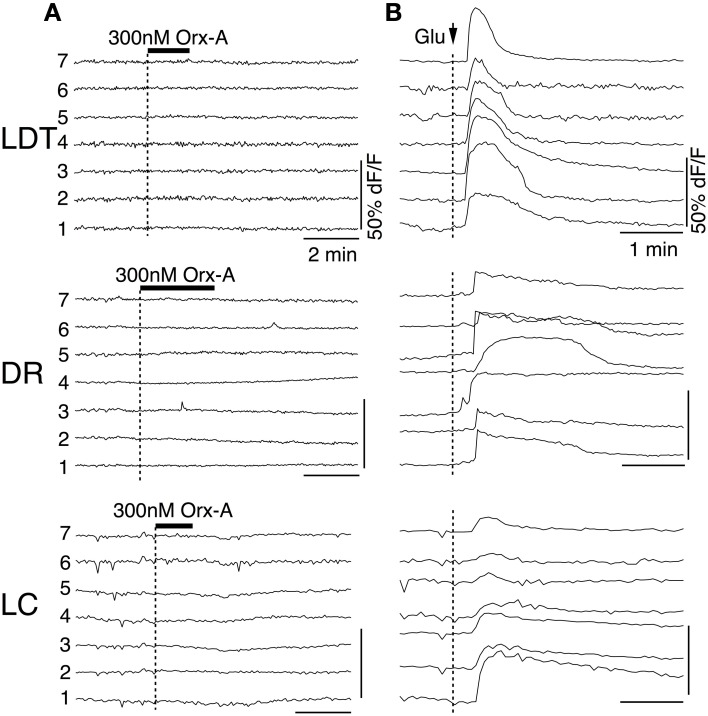
**Orexin fails to produce Ca^2+^ transients in slices from DKO mice. (A)** Somatic dF/F signals recorded from fura-2AM loaded LDT, DR, and LC cells in slices from DKO mice. Orexin-A application did not produce detectable changes in dF/F for any recorded cell. (B) Bolus application of glutamate (5 mM; delivered at arrow) produced strong Ca^2+^ transients in the same cells indicating that they remained viable. Horizontal bars above the traces indicate application of orexin-A (Orx-A; 300 nM). Calibration bars indicate 50% dF/F and 2 min in **(A)** and 50% dF/F and 1 min in **(B)**.

Interpretation of our data from single receptor knockouts presupposes that there are only two orexin receptors responsible for orexin actions and that our test concentration of orexin-A is specific for these receptors. We directly tested this by examining the action of orexin-A on slices made from mice lacking both orexin receptors. As can be seen in Figure 10A, orexin-A (300 nM) did not evoke Ca^2+^ transients in slices from DKO mice. To verify that each of the cells recorded under these conditions remained viable, we followed orexin application with a bolus application of glutamate (5 mM), which rapidly and effectively evoked Ca^2+^ transients in these same cells (Figure 10B). We also examined extracellular recordings using cell-attached patch recordings, whole-cell currents and the ability of orexin to augment the Ca^2+^ transients produced by step depolarizations to −30 mV. In each of these tests, orexin-A failed to produce a response. Collectively these data strongly indicate that 300 nM orexin-A is specific for native orexin receptors and that OX_1_ and OX_2_ are the only functional orexin receptors expressed in the LDT, DR and LC.

### Whole brainstem OX_2_ mRNA levels are higher in mice lacking OX_1_

**Figure 11:**
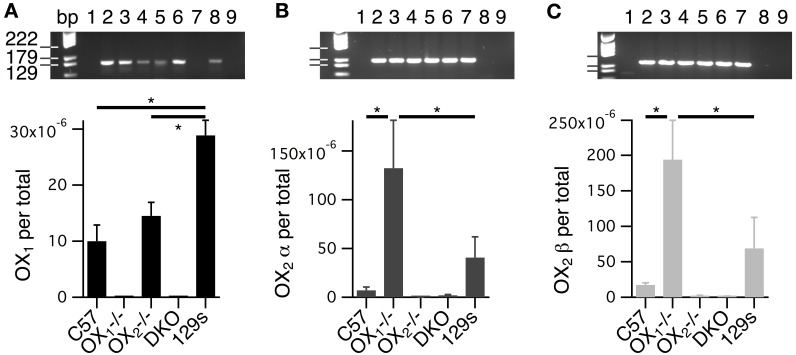
**Levels of whole brainstem OX_2_ mRNA are higher in OX^−/−^_1_ mice compared to wild-type mice. (A–C)** Top Gel images of the amplicons resulting from probes for OX_1_ **(A)**, OX_2_α **(B)**, and OX_2_β **(C)**. Samples for each lane were 1: blank, 2–5: scales for corresponding receptor, 6: C57 sample, 7: OX^−/−^_1_ sample, 8: OX^−/−^_2_ sample, 9: DKO sample. **(A)** Bottom RNA for OX_1_ (OX_1_ per total) was quantified from whole brainstems of C57BL6(C57; 28 samples from 14 mice), OX^−/−^_1_ (9 samples from 5 mice), OX^−/−^_2_ (28 samples from 14 mice), double orexin receptor knockouts (DKO; 8 samples from 4 mice) and 129SvEv (129s; 16 samples from 8 mice) mice by RT-PCR. OX_1_ mRNA was undetectable in tissue from OX_1−/−_ and DKO mice. Despite significantly higher levels of OX_1_ mRNA in brainstems from 129s mice compared to C57 and OX^−/−^_2_ brainstems (^*^P < 0.0001), there was no significant difference between mRNA levels measured in brainstems from C57 and OX^−/−^_2_ mice. **(B)** Bottom mRNA levels for OX_2_α (OX_2_α per total) were measured from isolates obtained from the same mice used in **(A)**. OX_2_α mRNA was undetectable in tissue from OX^−/−^_2_ (7 samples from 4 mice) and DKOs (20 samples from 10 mice). OX_2_α mRNA levels were higher in OX^−/−^_1_ samples (20 samples from 10 mice) than in either C57 (14 samples from 7 mice) or 129s (16 samples from 8 mice) samples (^*^P < 0.02). **(C)** Bottom Similarly, mRNA levels for OX_2_β (OX_2_β per total) were measured from mRNA isolates from the same mice in A. OX_2_β mRNA was undetectable in tissue from OX^−/−^_2_ (14 samples from 7 mice) and DKOs (20 samples from 10 mice). OX_2_β mRNA levels were higher in tissue from OX^−/−^_1_ mice (26 samples from 13 mice) than in tissue from either C57 (14 samples from 7 mice) or 129s mice (15 samples from 8 mice, ^*^P < 0.02).

A potential complication to the interpretation of data from constitutive knockouts is the possibility that the loss of one receptor alters the expression of the other. Indeed, like any lesion study, knockouts can't reveal the function of the missing component but can only reveal the capacity remaining in the absence of that component. We therefore compared OX_1_ and OX_2_ mRNA levels isolated from whole brainstems of C57BL6 and receptor knockout mice using quantitative RT-PCR to (Figure 11). Since two splice variants of the OX_2_ were identified in mice (Chen and Randeva, 2004; Chen et al., 2006), we designed primers for OX_1_ and both OX_2_ receptors. The primers used for each receptor were specific since PCR produced single amplicons of the predicted sizes. These amplicons were undetectable in samples from the corresponding single receptor knockout or double receptor knockouts (Figure 11, see gel insets). Results from ANOVAs comparing target mRNA levels by genotype were highly significant for each transcript (*P* < 0.001). *Post-hoc* testing revealed that the fraction of OX_1_ mRNA per total mRNA in brainstems from C57BL6 mice (9.75E-6 ± 2.00E-6, *n* = 28 samples from 14 mice) was not different from that measured from OX^−/−^_2_ brainstems (1.07E-5 ± 1.39E-6, *n* = 28 samples from 14 mice; *P* = 0.62; Figure 11A). In contrast, we found that in OX^−/−^_1_ mice, levels of both the OX_2_α (9.48E-5 ± 2.52E-5, *n* = 20 samples from 10 mice) and OX_2_β (1.88E-4 ± 3.97E-5, *n* = 26 samples from 13 mice) splice variants were significantly higher compared to those from C57BL6 mice (OX_2_α: 7.16E-6 ± 2.27E-6, *n* = 14 samples from 7 mice, *P* < 0.0001; OX_2_β: 1.94E-5 ±3.78E-6, *n* = 14 samples from 7 mice, *P* < 0.0005; Figures 11B,C).

Since these differences could indicate upregulation of OX_2_ expression resulting from the absence of OX_1_ and since the single receptor knockouts were on a mixed C57BL6 and 129SvEv background, we also measured receptor levels in 129SvEv mice. This comparison indicated that levels of OX_1_ are higher in 129SvEv brainstems than in either C57BL6 or OX^−/−^_2_ brainstems (2.89E-5 ± 2.04-06, *n* = 16 samples from 8 mice, *P* < 0.0001 for both; Figure 9A). Interestingly, levels of OX_2_α (4.08E-5 ± 1.46E-5, *n* = 16 samples from 8 mice) and OX_2_β (7.21E-5 ± 3.22E-5, *n* = 15 samples from 8 mice) from 129SvEv brainstems were not statistically different from those in C57BL6 brainstems (OX_2_α: *P* = 0.18; OX_2_β: *P* = 0.17) but were significantly lower than those from OX^−/−^_1_ brainstems (OX_2_α: *P* < 0.02; OX_2_β: *P* < 0.02; Figures 11B,C). These findings suggest that background alone is not the reason that OX_2_ mRNA levels are higher in brainstems from OX^−/−^_1_ mice and therefore imply some level of compensation.

## Discussion

A major finding of this study is that OX_1_ exclusively mediates direct depolarization of nNOS+ LDT and TH+ LC neurons, while both receptors mediate direct depolarization of TpH+ DR neurons. In contrast, augmentation of depolarization-induced Ca^2+^ transients was mediated by OX_1_ and OX_2_ in each nucleus and likely involved L-type Ca^2+^ channels and PKC signaling. Finally, we found whole-brainstem OX_2_ mRNA levels were elevated in OX^−/−^_1_ mice. These findings have implications for understanding the cellular function of native orexin receptors, for understanding the roles played by orexin signaling at these loci in the control of behavioral state and for understanding the consequences of using receptor specific antagonists as therapeutics.

Interpretation of our results are predicated on the idea that the different observed actions of orexin in OX_1_ null and OX_2_ null slices result from the absence of orexin receptors rather than from differences in genetic background between each mouse. Since genetic drift occurs and there is allelic variation in each parent strain, possibly in modifier genes, there is some uncertainty to this interpretation (Doetschman, 2009). We found that orexin currents had the same variation of responses and mean amplitude in both C57BL6 mice and in OxrWT, which have the same mixed genetic background as our knockout mice. In both strains, slices from each mouse showed responses to orexin and neither strain showed symptoms of narcolepsy (Kalogiannis et al., 2011) indicating that despite genetic drift and the allelic variations present, orexin signaling at our targets was equivalent in both backgrounds. Moreover, in DKO mice there were no response in any of the cells recorded in any mouse tested, and these mice show severe signs of narcolepsy (Kalogiannis et al., 2011). These considerations, and the recent evidence that the distribution of orexin receptor mRNA is not altered in LDT, DR, or LC in orexin receptor knockouts (Mieda et al., 2011) suggest it is unlikely that the absence of orexin responses result from background effects rather than the loss of the receptor. We therefore interpret our data in terms of receptor loss, mindful of the possibility that responses might also be modulated by background effects.

### Both native orexin receptors can couple to a noisy cation current and L-type Ca^2+^ channels

Recordings from DR neurons in slices from mice lacking either OX_2_ or OX_1_ revealed that activation of either receptor evoked a noisy inward current appearing identical to the cation current evoked in wild-type slices (Brown et al., 2002; Liu et al., 2002; Kohlmeier et al., 2008). Since orexin-A did not activate this current in slices lacking both receptors, we conclude that signaling by either receptor converges onto this current and that there are no additional orexin-binding receptors sufficient to mediate this action. The ability of both receptors to augment depolarization-induced Ca^2+^ transients mediated by L-type Ca^2+^ channels via a PKC-sensitive pathway further supports convergent or redundant functions of native OX_1_ and OX_2_ in DR neurons. Immunofluorescence identification demonstrated that this convergence includes serotonergic DR neurons (TpH+). These findings fit well with double *in-situ* hybridization evidence indicating both receptors are co-localized within a large fraction of TpH-expressing neurons in mouse DR (Mieda et al., 2011), and with single-cell RT-PCR evidence indicating both OX_1_ and OX_2_ mRNA is typically recovered from TpH neurons in rat (Brown et al., 2002). Together, these data suggest that both orexin receptors normally excite TpH neurons and augment their intracellular Ca^2+^-levels during periods of prolonged depolarization.

Since the average OX_1_–evoked inward current was somewhat larger than the OX_2_–evoked current, and since OX_1_ activation produced Ca^2+^ transients in a greater fraction of DR neurons than did OX_2_ activation, it is plausible that OX_1_ normally mediates the larger part of the orexin-mediated depolarization of DR neurons. A previous pharmacological study of rat DR slices concluded that OX_2_ is primarily responsible for orexin-evoked spiking (Soffin et al., 2004). However, that conclusion was based on data that was acknowledged to be difficult to interpret and suffered from limitations related to the use of selective agonists for receptor subtype identification (see also Leonard and Kukkonen, 2013). Co-expression and convergent action of these receptors also likely contributed to interpretive difficulties (e.g., the relatively weak effect of the OX_1_-selective antagonist SB 334867). Use of conditional receptor knockouts and development of subtype-specific receptor antagonists should help to further clarify the natural role of each receptor.

In contrast to the DR, OX_1_ was necessary for orexin to elicit depolarizing currents in principal LDT and LC neurons, which fits well with the high levels of OX_1_ mRNA in these structures from adult rodents (Marcus et al., 2001; Mieda et al., 2011). Since OX_2_ is competent to activate a similar depolarizing current in DR neurons, it is possible that OX_2_ is either not expressed, or is incapable of activating available depolarizing channels in LDT and LC neurons. Isotopic *in-situ* hybridization indicates OX_2_ message is much less abundant than OX_1_ but is above background in both the LDT and LC (Marcus et al., 2001), suggesting that low levels of OX_2_ are present. Recent double, non-isotopic, *in-situ* hybridization studies detected OX_2_ only in non-cholinergic LDT neurons and non-TH+ LC neurons (Mieda et al., 2011). Nevertheless, we found clear examples of LDT and LC neurons where orexin-A enhanced depolarization-induced Ca^2+^ transients in OX_1_ null slices, albeit fewer LDT and LC neurons were activated compared to slices from wild-type or OX_2_ null mice (Figure 8). Moreover, we recovered nNOS+ and TH+ neurons showing orexin-augmented Ca^2+^-transients, indicating that OX_2_ signaling influences at least a sub-population of these neurons (Figure 6). These latter findings are consistent with the possibility that OX_2_ expression in LDT and LC neurons might be developmentally regulated and could be lower in adult mice compared to the 2–4 week-old-mice used here, or simply that functional OX_2_ receptors are present, but that mRNA levels are too low to be detected with double *in-situ* hybridization methods, which are generally less sensitive than isotopic methods. If this is the case, how then does OX_2_ augment the depolarization-induced Ca^2+^ influx without also producing a depolarization? One possibility is that there is spatial segregation of OX_1_ and OX_2_ and their respective effectors such that OX_2_ signaling can't activate the depolarization channels. Another possibility is that each receptor activates different second messenger cascades and the depolarizing channels in LDT and LC are only activated by the cascade(s) from OX_1_. Neither scenario has been demonstrated for native orexin receptors and both deserve further investigation. In general, the messenger cascades underlying native orexin receptor actions are poorly understood, although they appear more diverse that originally thought (for review see Kukkonen and Leonard, 2013). Moreover, the cascades mediating depolarization in these neurons have not been identified, although since PKC is involved with augmenting Ca^2+^-transients, both receptors may activate PLC, as has been often found elsewhere (for review see Kukkonen and Leonard, 2013). While the cation currents activated in LDT and DR appear quite similar, we did find previously that low-Ca^2+^ ACSF augments the DR current, but not the LDT current (Kohlmeier et al., 2008), suggesting the possibility that different channels may mediate the orexin-activated cation current in these neurons. Future experiments are needed to clarify the signaling pathways and effectors utilized by each receptor in these neurons.

Finally, it is also possible that the orexin-enhanced Ca^2+^ transients were produced indirectly by an unknown mediator released from OX_2_-expressing nNOS- and TH- neurons in response to orexin-A. This seems unlikely since blocking action potentials with TTX, did not attenuate the orexin effects. However, this is difficult to rule out since conventional procedures to block Ca^2+^-dependent release, like lowering extracellular Ca^2+^ or adding Co^2+^ or Cd^2+^, also block the Ca^2+^ influx we monitored. Future studies using acutely isolated neurons could help address this issue.

### Roles of orexin signaling in reticular activating system neurons

Our main finding demonstrates that OX_1_ is required for orexin-mediated excitation of LC and LDT neurons but that either receptor is sufficient to mediate excitation of DR neurons. While subtle developmental changes in orexin receptor distribution cannot be ruled out, this main finding, obtained from young mice, agrees very well with orexin receptor mRNA levels in these neurons from adult mice (Mieda et al., 2011). It is therefore possible to compare the impairment of excitation observed here to the degree of behavioral impairment reported for each knockout, in order to gain insight into the functional consequences of orexin receptor signaling at these loci. Deletion of both receptors produces a narcolepsy phenotype (Hondo et al., 2010; Kalogiannis et al., 2011) similar to that seen in the prepro-orexin knockouts (Chemelli et al., 1999; Mochizuki et al., 2004), with fragmented waking states, spontaneous sleep attacks and frequent cataplexy. Since orexin-A normally stimulates a large fraction of LDT, DR, and LC neurons, the loss of orexin action at these sites in the DKOs is consistent with a role for orexin signaling at these loci in promoting wake and sleep consolidation, suppressing REM sleep, preventing sleep attacks and suppressing cataplexy. Indeed, optogenetic excitation of orexin neurons promotes sleep-to-wake transitions (Adamantidis et al., 2007) and optogenetic inhibition of orexin neurons promotes slow-wave sleep and reduced firing of DR neurons (Tsunematsu et al., 2011), while focal orexin injection into the LDT (Xi et al., 2001) or LC (Bourgin et al., 2000) prolongs waking bouts and suppresses REM sleep. Moreover, optogenetic inhibition of TH+ LC neurons blocks the ability of orexin neuron stimulation to promote sleep-to-wake transitions (Carter et al., 2012), while ontogenetic stimulation of TH+ LC neurons both prolongs waking (Carter et al., 2010) and enhances the wake-promoting effect of orexin neuron stimulation (Carter et al., 2012). This indicates orexin–excitation of TH+ LC neurons is necessary for orexin neuron activity to promote sleep-to-wake transitions. Nevertheless, it is the OX_2_ knockouts that show fragmented spontaneous waking and sleep states and have sleep attacks at the same frequency as prepro-orexin knockouts (Willie et al., 2003), even though orexin-mediated excitation of LDT, DR, and LC neurons is preserved (Figure 2). This might indicate that orexin neuron firing and orexin release is impaired through the loss of OX_2_-mediated positive feedback (Yamanaka et al., 2010) and/or that residual OX_1_-excitation of LDT, DR, and LC neurons is insufficient to maintain normal duration bouts of spontaneous waking and sleep in the absence of OX_2_, even though ICV orexin still prolongs waking and suppresses REM sleep in OX^−/−^_2_ mice (Mieda et al., 2011). Conversely, OX^−/−^_1_ mice appear to have normal sleep-wake states without fragmentation or sleep attacks (Hondo et al., 2010; Mieda et al., 2011), even though orexin excitation is abolished in LDT and LC neurons and is reduced in DR neurons (Figure 5), in spite of possible upregulation of OX_2_ (Figure 11). Thus, it appears intact orexin excitation in the LDT, DR, and LC is neither necessary nor sufficient for the expression of normally consolidated bouts of spontaneous sleep and waking. Since, OX_1_ signaling in LDT, DR, and LC promotes arousal, but is not necessary for consolidated spontaneous waking, orexin signaling at these loci may promote context-dependent arousal associated with, for example, stress, anxiety, panic, and/or food and drug seeking behavior (Winskey-Sommerer et al., 2004; Boutrel et al., 2005; Harris et al., 2005; Nair et al., 2008; Johnson et al., 2012; Piccoli et al., 2012; Steiner et al., 2012; Heydendael et al., 2013). Orexin signaling at these loci may also support other functions. For example, OX^−/−^_1_ mice show impaired acquisition and expression of cued and contextual fear conditioning and the re-expression of OX_1_ in TH+ LC neurons rescues the expression of cued fear (Soya et al., 2013). Future studies using receptor rescue approaches (Mochizuki et al., 2011) or the local application of OX_1_-specific antagonists will be needed to further explore OX_1_ functions at these loci.

Our findings are also consistent with a cataplexy-suppressing role for orexin excitation in the LDT, DR and LC. OX^−/−^_2_ mice have cataplexy but it occurs rarely compared with prepro-orexin null mice (Willie et al., 2003). Since DKO mice show frequent cataplexy-like arrests (Kalogiannis et al., 2011), the less severe cataplexy in OX^−/−^_2_ mice is likely related to residual OX_1_ signaling. Since orexin injections into the LDT and LC suppress REM sleep, and knockdown of OX_1_ in LC increases REM during the dark phase (Chen et al., 2010) it is plausible that remaining OX_1_-excitation in LDT, LC, and perhaps DR, reduces cataplexy in OX^−/−^_2_ mice. Nevertheless, OX^−/−^_1_ mice do not have cataplexy, indicating that the loss of orexin-excitation in LDT and LC and reduced orexin excitation in the DR is not sufficient to produce cataplexy, at least in these knockouts, where OX_2_ may also be up regulated.

### Implications for single orexin receptor pharmacotherapeutics

DORAs show significant promise for improved treatment of insomnia (Uslaner et al., 2013; Winrow and Renger, 2013) and are currently being considered for FDA approval. Given that orexins regulate numerous functions beyond normal waking and sleep and that their receptors are differentially distributed, it seems likely that subtype-specific orexin receptor antagonists (SORAs) will have even greater therapeutic potential. The development of sub-type specific drugs will accelerate preclinical investigations of receptor function and, may allow better targeting of different orexin-dependent behaviors and a fine-tuning of their sleep-promoting effects. For example, OX_2_ antagonists appear more effective at sleep promotion than DORAs (Dugovic et al., 2009), perhaps by targeting hypothalamic circuits promoting histamine release and consolidated waking (Dugovic et al., 2009; Mochizuki et al., 2011). Conversely, OX_1_ antagonists may provide better relief for hyperarousal and other maladaptive behaviors associated with stress, as noted above, perhaps by dampening OX_1_ excitation of noradrenergic, serotonergic and cholinergic reticular neurons and midbrain dopamine systems. Nevertheless, much more needs to be learned about how each orexin receptor impacts their cellular targets and how these targets influence behavior. These interactions are likely to be complex: as demonstrated here for key elements of the reticular activating system, each receptor can have common effectors (i.e., both receptors activating depolarizing channels and enhancing voltage-dependent Ca^2+^ transients in TpH DR neurons) or act differentially with respect to one effector while having a common action on another effector (i.e., OX_1_ is necessary for depolarization while both receptors enhance voltage-dependent Ca^2+^ transients in LDT and LC neurons). It will also be important in future experiments to evaluate the degree to which persistent alteration of signaling by either orexin receptor impacts synaptic plasticity (Borgland et al., 2006; Selbach et al., 2010), neurotransmitter phenotypes (Kalogiannis et al., 2010; Valko et al., 2013) and orexin receptor expression (i.e., elevated brainstem OX_2_ message levels in OX_1_ null mice), as each of these could lead to deleterious side-effects and diminished therapeutic potential.

### Conflict of interest statement

The authors declare that the research was conducted in the absence of any commercial or financial relationships that could be construed as a potential conflict of interest.
